# Positive psychological well-being and cardiovascular health

**DOI:** 10.3389/fpsyt.2024.1443978

**Published:** 2024-10-25

**Authors:** Claudia Zuccarella-Hackl, Mary Princip, Sinthujan Sivakumar, Roland von Känel

**Affiliations:** Department of Consultation-Liaison Psychiatry and Psychosomatic Medicine, University Hospital Zurich, University of Zurich, Zurich, Switzerland

**Keywords:** positive psychological well-being, hedonic well-being, eudaimonic well-being cardiovascular health, biological mechanisms, intervention studies

## Abstract

Positive psychological well-being (PPWB) is increasingly recognized as a critical factor in cardiovascular health of both healthy individuals and those with cardiovascular diseases (CVD). This mini-review synthesizes the current state of knowledge on the relationship between PPWB and cardiovascular health, examining relevant studies on PPWB in both populations. The conceptualization of PPWB encompasses hedonic and eudaimonic facets, with constructs such as optimism, purpose in life, and vitality playing crucial roles. Studies among healthy individuals show a significant association between PPWB and improved cardiovascular health indicators, while research among cardiac patients highlights the importance of PPWB in predicting outcomes such as mortality and rehospitalization. Mechanistic pathways linking PPWB and cardiovascular health include biological processes, health behavior changes, and additional psychological resources that mitigate stress. Despite the growing evidence, questions remain unanswered, necessitating further research to understand these relationships and develop effective interventions. Promoting psychological well-being alongside physical health can enhance cardiovascular disease prevention and management, offering a comprehensive approach to improving patient outcomes and overall well-being.

## 1 Introduction

According to the World Health Organization, mental health (WHO) is described as “a state of well-being where individuals are able to fulfill their potential, effectively manage everyday stresses, maintain productive work, and contribute to their community (1).” Negative psychological health encompasses conditions like depression, chronic stress, anxiety, anger, pessimism, and overall life dissatisfaction. Conversely, positive psychological health entails traits such as optimism, a sense of purpose, gratitude, resilience, positive emotions, and happiness (2).

Positive psychological well-being (PPWB) is increasingly acknowledged as a crucial factor in cardiovascular health, counterbalancing traditionally emphasized risk factors like stress, depression, anger and anxiety (3). Encompassing elements such as happiness, optimism, resilience, life satisfaction, and a sense of purpose, PPWB significantly impacts cardiovascular health and overall cardiovascular function (3–5). Research indicates that positive psychology factors, including well-being, optimism, and positive affect, are prospectively linked to a lower risk of mortality and rehospitalization in patients with cardiovascular disease (CVD) (6). Overall, the strongest evidence was found for optimism (7). This may be partly due to the fact that more long-term cohort studies have assessed optimism, while fewer epidemiological studies have measured other aspects of psychological well-being (8).

It is important to note that positive psychological constructs are not merely the opposite of negative psychological states. Positive and negative constructs, such as optimism and depression, show only a modest inverse correlation (e.g., r = -0.3) (9). Moreover, the links between positive psychological well-being and health outcomes are often maintained even when accounting for the effects of depression (10). This indicates that the health benefits associated with positive emotions and thoughts are not simply due to the absence of depression.

With growing interest in the connections between positive psychological constructs and health, several literature reviews have been published[ (2, 11, 12). However, several questions in this field remain unanswered, particularly regarding the specific mechanisms through which positive psychological well-being influences cardiovascular health. Research gaps include the need for more longitudinal studies to establish causal relationships, a better understanding of the biological pathways involved, and clarification of how different aspects of psychological well-being, like optimism and life satisfaction, affect cardiovascular outcomes. Evidence is also lacking or inconclusive on whether interventions to enhance psychological well-being can directly improve cardiovascular health (13). This Mini-Review aims to summarize current knowledge about the relationship between PPWB and cardiovascular health drawing on relevant studies focusing on systematic reviews and meta-analyses and large-scale cohort studies. The mini-review is divided into six sections: conceptualization of PPWB; PPWB and cardiovascular health; mechanisms linking PPWB and cardiovascular health; potential clinical implications, limitations and future directions for research. Its objectives are to summarize key findings on the relationship between psychological well-being and cardiovascular health, identify gaps in understanding how positive psychological factors affect cardiovascular outcomes, and clarify relevant concepts. The study also highlights promising areas for further exploration, such as the impact of stress reduction and positive psychological interventions on cardiovascular health.

## 2 Conceptualization of positive psychological well-being

**Figure 1:**
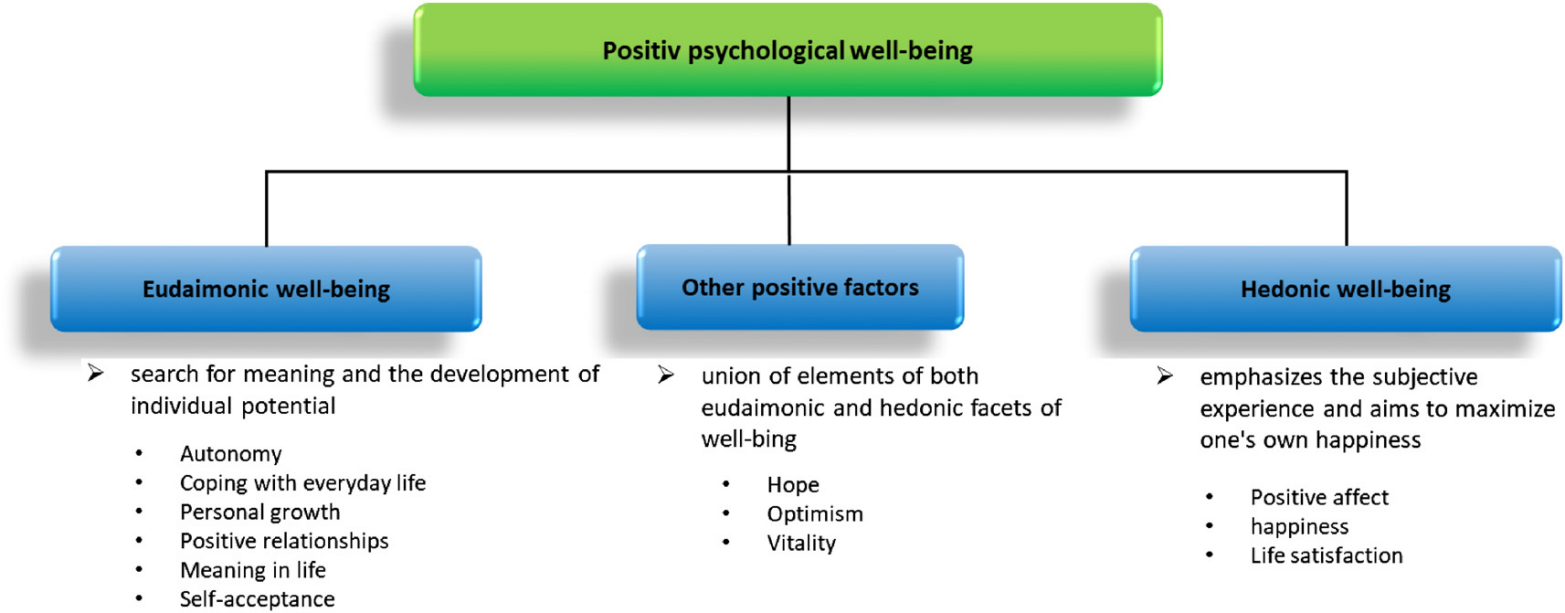
Conceptualization of positive psychological well-being. Adapted from (3, 11, 14).

In current positive psychology research, various variables are being intensively investigated. Due to a considerable degree of overlap among positive psychological constructs such as happiness, optimism, and life satisfaction, debates persist regarding their definitions and distinctions. This lack of consensus complicates research into their specific effects on cardiovascular health (11, 12, 14, 15). One possible theoretical approach is the concept of well-being (16, 17). Fundamentally, psychological well-being encompasses positive thoughts and feelings that people use to evaluate their lives as positive (14). Specifically, psychological well-being includes positively evaluated feelings and cognitive assessments. It is often defined as an umbrella term for various aspects of positive psychology. It can include dimensions such as meaning in life, positive affect, life satisfaction, and optimism (3, 8, 14). Well-being can be divided into hedonic and eudaimonic well-being based on philosophical considerations (14). Hedonistic well-being emphasizes the subjective experience and aims to maximize one’s own happiness. Eudaimonic well-being, on the other hand, focuses on the search for meaning and the development of individual potential. While the former consists of the components life satisfaction, positive affect and happiness, the latter includes autonomy, coping with everyday life, personal growth, positive relationships, meaning in life and self-acceptance (14, 17, 18). The concept has since been expanded. Newer versions propose additional subtypes that do not clearly fit into either of the two facets of well-being. Due to their combination of elements from both the eudaimonic and hedonic facets, three constructs - hope, optimism, and vitality - could be implemented as a third category (14) (see **Figure 1**).

## 3 Positive psychological well-being and cardiovascular health

Cardiovascular health refers to the functionality of the heart and blood vessels, ensuring efficient delivery of oxygen and nutrients to tissues and removal of waste products. The American Heart Association (AHA) defines cardiovascular health broadly, including more than just the absence of disease (19). It aims to improve cardiovascular health for all through seven key behaviors and factors: diet, physical activity, nicotine exposure, body mass index, fasting blood glucose, blood lipids, and blood pressure (BP). These are categorized as poor, intermediate, or ideal, with ideal health requiring all seven at optimal levels. Recently, sleep behavior has been added to this concept (20). Cardiovascular health is often assessed by outcomes like cardiovascular disease (e.g., myocardial infarction) and mortality. Neurobiological aspects are crucial in understanding cardiovascular health, primarily due to the intricate interactions between the nervous system and the cardiovascular system (21). For example, the autonomic nervous system regulates heart rate and BP, while chronic stress can induce inflammation and increase cardiovascular risk through the hypothalamic-pituitary-adrenal (HPA) axis. Neurotransmitters and bilateral communication between the heart and brain also play pivotal roles in cardiovascular health. Additionally, mental health and social factors should be considered within the framework of cardiovascular health and neurobiological mechanisms (21). In recent years, numerous publications, especially systematic reviews and meta-analyses, have explored the relationship between positive psychology constructs like well-being and various cardiovascular health indicators (e.g. 2, 11, 12, 14). While some studies have focused on understanding these associations among individuals with CVD, others have turned their focus on the interplay between positive psychological constructs and cardiac health outcomes in healthy populations. In the subsequent chapters, we will first examine findings from studies conducted among healthy individuals, followed by a brief summary of research involving cardiac patients. The two sections aim to provide a comprehensive understanding of the overlap between positive psychology and cardiovascular health across a spectrum of these two populations.

### 3.1 Healthy individuals

Boehm and colleagues found a significant link between PPWB and better cardiovascular health in older adults (≥50 years) from the English Longitudinal Study of Aging. Indicators included cholesterol, blood pressure (BP), body mass index (BMI), diabetes, and smoking history. Higher psychological well-being correlated with a 29% reduced risk of CVD (22). Another study on young adults (18–30 years) showed that greater baseline positive emotions was linked to better cardiovascular health over 20 years, even after accounting for various factors (23).

In a large-scale study with 1,739 participants, elevated positive affect was associated with a decreased risk of coronary heart disease over a decade, independent of other factors (4). A meta-analysis of 136,265 individuals found that a higher sense of purpose in life was associated with a reduced risk of incident cardiovascular events (24). Recently, cognitive constructs such as life satisfaction have gained attention. A study involving 6,251 participants found that higher life satisfaction correlated with lower blood vessel calcification, but this association disappeared after adjusting for cardiovascular risk factors (25).

### 3.2 Patients with cardiovascular disease

In the context of cardiac patients, positive psychology constructs are often examined in relation to outcomes such as rehospitalization, reinfarction, or death. A review by DuBois et al. (6) found that two studies indicated heightened psychological well-being correlates with reduced mortality following coronary artery disease (CAD) and fewer adverse events such as myocardial infarction. Additionally, a review of 113 studies found that eudaimonic well-being such as purpose in life, was linked to improved overall mental health following a cardiovascular event. Patients with a sense of purpose in life experience reduced stress and show favorable physiological biomarkers (e.g. C-reactive protein (CRP), BP) (26). Conversely, an existential crisis or difficulty finding purpose after such an event is associated with poorer physical health, lower quality of life, decreased motivation for lifestyle changes, increased psychological stress, and higher cardiovascular risk (26). Roepke et al. (27) noted in their systematic review, that patients possessing a sense of purpose in life tend to experience accelerated recovery following myocardial infarction. An increasing number of studies are emerging that link optimism to various cardiovascular parameters within the framework of positive psychology research. In a recent review, Amonoo et al. (28) support the finding that optimism is prospectively linked to better outcomes, such as fewer reinfarctions. Here, too, the authors conclude that health behavior play a decisive role. Regarding the link between psychological well-being and cardiovascular outcomes, various pathways - biological, behavioral, and psychosocial - could play an important role (8). For example, the relationship between psychological well-being and longer survival in patients with CVD could be explained by better medication adherence (29).

Overall, these findings support the notion, that psychological well-being should be prioritized not only in clinical settings, but also across the entire population, both healthy and diseased. These findings highlight the importance of understanding the underlying mechanisms and mediators. Sin and colleagues reached a similar conclusion, stating that overall psychological well-being can serve as a protective factor against CVD based on extensive studies (30). In their review, they discuss the extensive positive effects on the immune, neuroendocrine and cardiovascular functioning. They also address the benefits such as the reduced stress reactivity and enhanced coping skills resulting from better well-being. Importantly, they conclude that health behaviors may serve as crucial mediators between well-being and CVD.

## 4 Mechanisms linking positive psychological well-being to cardiovascular health

Numerous review articles suggest that PPWB influences cardiovascular health through three potential underlying pathways, all of which reduce deteriorative and/or enhance restorative processes: 1) a direct pathway through biological processes, 2) an indirect pathway through health behaviors, and 3) the promotion of additional psychological resources has been found to preserve health and mitigate detrimental effects of stress (2, 8, 11, 12). Neurobiologically, PPWB has been associated with various brain regions and neurotransmitters, including (nor)epinephrine and serotonin. Nevertheless, these associations have shown inconsistency, with neurotransmitter levels often demonstrating little correlation with measures of PPWB (31, 32).

Potential behavioral factors that may mediate the relationship between PPWB and cardiovascular health include adopting a healthier lifestyle (33), such as increased physical activity, reduced smoking, improved diet (34), better sleep quality and duration (35), decreased sleep disturbances (36), and enhanced medication adherence (29, 37). Additionally, psychological resources can mitigate the impact of stress and encompass improved social support (38), adaptive coping mechanisms (39), better emotional and behavioral regulation (40), and higher sociodemographic factors (18).

### 4.1 Biological pathway

#### 4.1.1 Literature search

In the following section, we summarize findings from the past 5 years on the relationship of PPWB and the biological pathways in both healthy individuals and cardiac patients, with a particular focus on their relevance to cardiovascular disease. To provide a comprehensive overview, we particularly focused on evidence from large observational studies, both longitudinal and cross-sectional, randomized controlled trials, scientific statements and systematic reviews and meta-analyses. Research has shown that various constructs of PPWB are associated with both restorative [e.g., heart rate variability (HRV)], and deteriorative (e.g. inflammation) physiological functions related to CVD (11). The literature search included published full-text articles from the databases MEDLINE and Google Scholar. For each database, the search strategy included terms reflecting PPWB and biomarkers relevant to CVD. PPWB search terms included variations of positive affect, happiness, life satisfaction, positive well-being, eudemonic and hedonic well-being, and optimism. Biomarkers included variations of inflammatory markers (e.g., CRP), cardiovascular risk factors (e.g., BP, lipids), and endocrine parameters (e.g., cortisol).

##### 4.1.1.1 Blood pressure

Research on the association between PPWB and BP yields mixed results. In a cross-sectional study, dispositional optimism was found to be negatively associated with systolic blood pressure (SBP) in relation to stressful events in undergraduates aged 18-24 years. No association was found with diastolic blood pressure (DBP) (41). In contrast, no association was found between optimism and both odds and ratio of nocturnal mean arterial blood pressure (MAP) dipping, as a restorative physiological process, in healthy participants of the North Texas Heart Study (42). However, in a prospective study of U.S. Soldiers, optimisms was associated with a 22% reduced risk of hypertension, independent of sociodemographic characteristics, and depression (43). A longitudinal study using data from the China Health and Retirement Longitudinal Study found life satisfaction was positively associated with high SBP after four years, although this association was not independent of depressive symptoms (44). Furthermore, no association was found between purpose in life and the risk of hypertension in US adults aged over50 years from the Health and Retirement Study (45).

##### 4.1.1.2 Heart rate*/*heart rate variability

Studies have indicated that PPWB can improve the regulation of the autonomic nervous system by measuring both heart rate (HR) and HRV. In a cross-sectional study of Bajaj et al. (41), dispositional optimism was negatively correlated with HR and in relation to stressful events. In a longitudinal study of 300 individuals recruited through medical practitioners, there was a strong association between positive affect and HRV. In addition, activities inducing positive affect (e.g. physical activity) were shown to be associated with higher HRV (46). Similar results were found by Hachenberger et al. (47), reporting a positive association between higher HR and HRV and positive affect at different time intervals. In a study using cognitive and psychological stress tasks participants with higher levels of positive affect showed higher HRV independent of sex, age, and baseline negative affect (48). In contrast, a longitudinal study analyzing HRV as a potential underlying mechanism for the association between optimism and reduced risk of CAD found no association between optimistic healthy aging women and HRV, nor any mediating effect for the association between optimism and CAD risk. (49). Similarly, a prospective analysis of data from the Mid-life in the United States study (MIDUS) revealed trait gratitude to be significantly associated with reduced risk of acute myocardial infarction after a follow-up of 6.7 years. This association was mediated by increased HR reactivity to stress (50).

##### 4.1.1.3 Lipids

Cross-sectional and longitudinal studies indicate an association between PPWB and lipids with, however, mixed results. A cross-sectional study in cardiac patients found an association between well-being (i.e. positive affect, positive relationships, positive functioning) and a better lipid-profile. Higher well-being was associated with lower levels of triglyceride, very low-density lipoprotein (VLDL), total cholesterol (TC) to high-density lipoprotein (HDL) ratio, and higher HDL levels (51). In contrast, in a study of 283 individuals over the age of 55, happiness was significantly and negatively associated with LDL only. For optimism and life satisfaction, no association was found with lipids (52). In participants of the MIDUS longitudinal study environmental mastery showed a significant negative association with sphingolipids (53), whereas higher dispositional gratitude was associated with lower triglyceride levels. The latter was partially mediated by a healthy diet and lower BMI (54). In addition, a longitudinal analysis in patients with a previous acute myocardial infarction showed a significant positive association between positive affect and HDL, and significant negative affect with TC/HDL ratio over time. These associations were independent of sociodemographic factors, indices of cardiac disease severity, comorbidity, medication use, health behaviors, serum cortisol, and negative affect (55). However, a study involving over 3,000 middle-aged individuals from the Coronary Artery Risk Development in Young Adults (CARDIA) study reported a significant negative association between optimism and both baseline TC and LDL but no association over time was found across different races (56).

##### 4.1.1.4 Arterial and aortic stiffness

There is some evidence that PPWB has an impact on aortic and arterial stiffness, as well as on blood vessel calcification. A cross-sectional study in cardiac patients found evidence for a negative association between well-being (i.e. positive affect, positive relationships, positive functioning) and arterial stiffness, independent of sociodemographic factors, and health-promoting behaviors (51). In more than 4,700 participants from the Whitehall II cohort study, higher eudaimonic well-being, measured as a sum score of all sub-facets of eudaimonic well-being, was associated with lower baseline aortic stiffness in men but not in women, independent of social, behavioral, and biological factors. This association persisted over 5 years. No such association was found for pleasure in life in either men or women (57). In a cross-sectional study involving more than 6,200 participants, higher life satisfaction has shown to be correlated with lower level of blood vessel calcification, a medical condition contributing to aortic and arterial stiffness. This association was independent of sociodemographic factors and depression, but not independent of cardiovascular risk factors (e.g. alcohol consumption, smoking) (25).

##### 4.1.1.5 Inflammation

Several cross-sectional and longitudinal studies examined the association between PPWB constructs and circulating inflammatory biomarkers. The results showed variation in these associations both within and between different types of studies. Over the past 5 years, there has been an increase in longitudinal studies examining this association with similar results. In participants from the Health and Retirement Study, purpose in life was prospectively and negatively associated with CRP, Interleukin (IL)-6, IL-10, Il-1ra, and soluble Tumor Necrosis Factor Receptor 1 (sTNFR1). These associations were not moderated by sociodemographic factors (58). In addition, purpose in life was negatively associated with the development of CRP levels indicating CVD risk among men, but not among women after a follow-up of 8 years (59). In the English Longitudinal Study of Ageing (ELSA),hedonic and eudaimonic well-being, specifically control-autonomy, was negatively correlated with CRP, fibrinogen, and white blood cells (WBC), whereas positive affect and life satisfaction were negatively associated with WBC, apparently independent of demographic and socio-economic factors (60). Two recent meta-analyses underscore these findings showing that PPWB (a composite index that integrates positive affect, positive relationships, positive functioning or life satisfaction, optimism, and happiness, respectively) was negatively associated with CRP and IL-6 levels (31, 61), while the association with IL-6 was not independent of covariates included in the reviewed studies (61). Other inflammatory markers, such as fibrinogen and Tumor-Necrosis-Factor (TNF)-α, were either negatively or non-significantly related to PPWB (31, 61).

##### 4.1.1.6 Additional biological parameters

Cortisol emerges as the hormone most extensively examined in relation to PPWB. A recent systematic review and meta-analysis revealed a significant negative correlation between PPWB (including sub-facets of eudaimonic well-begin, positive affect and happiness)and cortisol levels, as well as a more pronounced diurnal decline in cortisol levels. Inconsistent results were found for cortisol awakening response and area under the curve measures. The associations between other hormones (e.g. Dehydroepiandrosterone sulfate, testosterone, and vitamin D) were mainly non-significant (31). In recent cross-sectional studies examining leukocyte telomere length as a marker of cellular ageing, null associations were found with optimism across different races (62, 63).

## 5 Potential clinical implication

Given the data linking PPWB with CVD, the next step is to evaluate whether specific positive psychological interventions (PPI) can effectively modify PPWB and its underlying mechanisms (e.g. biological parameters) to improve cardiovascular health (8). In recent years, there has been a notable increase in the number of studies investigating PPIs and their effects on underlying mechanisms. A recent systematic review indicates that PPIs may improve behavior adherence (i.e. physical activity, medical adherence, diet, and smoking) in healthy individuals and patients with medical conditions (e.g. CVD, diabetes, hypertension). However, the effect size was rather small, with small sample sizes, generally low study quality, and inconsistent interventions and outcomes (64). To date, only three studies have investigated the effect of PPI on biological parameters. In two of these group-based PPI intervention studies conducted in cardiac patients, a significantly greater reduction in high-sensitive (hs)-CRP, fibrinogen, and lower cortisol awakening response was observed (65, 66), whereas one study found no effect on hs-CRP (67).

The potential clinical implications can manifest in different ways. First, according to the European Society of Cardiology Guidelines on CVD prevention in clinical practice (68), which recommend screening for psychological distress such as depression and anxiety, it might also be necessary to assess PPWB (2, 8). Brief questions about PPWB (e.g. optimism, positive affect, gratitude) (**Table 1**) can provide information and stimulate a conversation about promoting PPWB (2, 8). Second, patients can be advised to engage in hobbies or other enjoyable activities that include physical activity, social support, and increasing life satisfaction and meaning. Thirdly, implementation of specific structured PPWB-related activities including optimisms-focused activities linked to better PPWB (e.g. gratitude exercises, positive affirmations, positive reframing) can be considered. In a clinical setting that focuses on improving cardiovascular health (e.g. cardiac prevention and rehabilitation programs), there may be optimal opportunities to conduct these assessments, conversations, and interventions, which can prove to be effective and valued by patients. Moreover, clinicians with psychocardiology expertise should combine methods to improve well-being with other psychological or behavioral interventions (2, 8).

**Table 1:** Brief clinician questions to positive psychological well-being in clinical setting.

Positive well-being construct	Questions
Optimism	• “Do you expect that good things will happen for you in the future?” • “How do you think things will go with your health in the future?”
Positive affect or life satisfaction	• “How often do you experience pleasure or happiness in your life?” • “Are you satisfied with how your life has gone and how you have lived it?”
Gratitude	• “What, if anything, do you have to feel grateful for in your life?” • “Do you ever feel grateful about your health? Tell me about that.”

Adapted from (2, 8).

However, it should be noted that the relationship between PPWB and cardiovascular health is complex and influenced by various factors. Medical co-morbidities such as diabetes, hypertension, and obesity, along with psychiatric co-morbidities like depression and anxiety (69), and co-morbid addictive behaviors such as smoking (70), can all negatively influence both PPWB and cardiovascular health. Chronic stress, specific personality traits such as hostility and Type D, and maladaptive coping strategies further contribute to these adverse effects (2). Additionally, lifestyle behaviors as modifiable factors, and non-modifiable factors like genetics, age, and family history of CVD, also play significant roles (71). Understanding these interconnected factors is essential for developing integrative approaches aimed at enhancing both PPWB and cardiovascular health.

## 6 Limitation

Our mini-review has several limitations related to confounding factors and effect modifiers. Firstly, while the review synthesizes current knowledge, it may not comprehensively cover all aspects of PPWB and cardiovascular health due to constraints on depth and scope. Variations in study methodologies, including diverse measures of PPWB and populations studied, could introduce heterogeneity in findings, complicating the formulation of uniform conclusions. Additionally, the mini-review relies on existing systematic reviews, meta-analyses, and cohort studies, which vary in quality and rigor. Differences in study design, sample sizes, and controls for confounding factors may affect the reliability and generalizability of conclusions. Furthermore, factors such as socio-economic status, ethnicity, and health behaviors may interact with PPWB, influencing cardiovascular outcomes. Addressing these confounders is crucial for accurately interpreting the evidence. Future research should prioritize longitudinal studies, prospective observational studies across diverse populations of patients with CVD and settings. Additionally, further intervention studies are required to establish a comprehensive epidemiological model for elucidating the relationship between PPWB and cardiovascular health.

## 7 Future directions of research

Overall, it seems that well-being is prospectively associated with a reduced risk of cardiovascular events in both initially healthy individuals and cardiac patients. Cross-sectional and longitudinal research on the underlying biological pathways have found support for the association with PPWB but remains inconsistent. Additionally, different constructs of PPWB affect restorative and deteriorative parameters differently. To establish causality between psychological well-being and cardiovascular outcomes, more rigorous research into mechanistic pathways and genetic studies applying Mendelian Randomization (72, 73) would be crucial. Future investigations should employ longitudinal designs, incorporate multiple measures of PPWB, and examine various biological pathways simultaneously. Adequate control for confounders like psychological distress and large, diverse sample sizes are also essential. Studies of interventions that enhance PPWB should be a primary concern in both clinical practice and research. However, high-quality research is needed to determine the optimal intervention content, dosage, and delivery method to achieve effective and sustainable changes in the underlying mechanisms.

## 8 Conclusion

The results of the studies reviewed here provide much evidence for the significance of psychological well-being for cardiovascular health. A relationship between positive psychological constructs and improved health indicators has been found in both healthy individuals and patients with CVD. Despite the available evidence, several questions remain unanswered regarding the link between psychological well-being and cardiovascular outcomes. Future research should focus on investigating the mechanistic pathways to better understand these associations and develop appropriate interventions. Nonetheless, the influence of a positive attitude on cardiovascular outcomes highlights the importance of psychological interventions and lifestyle changes aimed at enhancing psychological well-being as part of a comprehensive approach to cardiovascular disease prevention and management. By promoting mental health alongside physical health, healthcare providers can better support their patients to achieve optimal cardiovascular outcomes and improved overall well-being.
